# Detection of LFM Radar Signals and Chirp Rate Estimation Based on Time-Frequency Rate Distribution

**DOI:** 10.3390/s21165415

**Published:** 2021-08-10

**Authors:** Ewa Swiercz, Dariusz Janczak, Krzysztof Konopko

**Affiliations:** Faculty of Electrical Engineering, Bialystok University of Technology, 15-351 Bialystok, Poland; d.janczak@pb.edu.pl (D.J.); k.konopko@pb.edu.pl (K.K.)

**Keywords:** LFM signal detection, parameter estimation, chirp signal, cubic phase function, chirp rate estimation

## Abstract

Linear frequency-modulated (LFM) signals are the most significant example of waveform used in low probability of intercept (LPI) radars, synthetic aperture radars and modern communication systems. Thus, interception and parameter estimation of the signals is one of the challenges in Electronic Support (ES) systems. The methods, which are widely used to accomplish this task are mainly based on transformations from time to time-frequency domain, which concentrate the energy of signals along an instantaneous frequency (IF) line. The most popular examples of such transforms are the short time Fourier transform (STFT) and Wigner-Ville distribution (WVD). However, for LFM waveforms, methods that concentrate signal energy along a line in the time-frequency rate domain may allow to obtain better detection and estimation performance. This type of transformation can be obtained using the cubic phase (CP) function (CPF). In the paper, the detection of LFM waveform and its chirp rate (CR) parameter estimation based on the extended forms of the standard CPF is proposed. The CPF was originally introduced for instantaneous frequency rate (IFR) estimation for quadratic frequency modulated (QFM) signals i.e., cubic phase signals. Summation or multiplication operations on time cross-sections of the CPF allow to formulate the extended forms of the CPF. Based on these forms, detection test statistics and the estimation procedure of LFM signal parameters have been proposed. The widely known estimation methods assure satisfying accuracy for high SNR levels, but for low SNRs the reliable estimation is a challenge. The proposed approach based on joint analysis of detection and estimation characteristics allows to increase the reliability of chirp rate estimates for low SNRs. The results of Monte-Carlo simulation investigations on LFM signal detection and chirp rate estimation evaluated by the mean squared error (MSE) obtained by the proposed methods with comparisons to the Cramer-Rao lower bound (CRLB) are presented.

## 1. Introduction

LPI radars are a class of radar systems that are designed to be difficult to detect by today’s electronic support measures (ESM) [1]. The LPI radar transmits a low power intrapulse modulated waveform or frequency modulated continuous wave (FMCW) to reduce the transmitted power of LPI waveforms. LFM signals are important signals among LPI signal modulations. Such signals are addressed in Electronic Intelligence (ELINT) which collect and process weak signals, especially radar signals, allowing tactical action to recognise radiation sources and the type of signals modulation for effective counteraction [2,3,4] in today’s battlefields. The growing interest in Passive Coherent Location (PCL) systems with non-cooperative transmitters, such as those operating in ultra high frequency (UHF) and very high frequency (VHF) bands, forces incrisingly advanced analysis of frequency modulated (FM) signals, including LFM signals [5]. LFM signals belong to the class of polynomial phase signals, which are intensively used in imaging of moving and non-moving targets in modern synthetic aperture radar (SAR) imaging systems [6]. The rapid development of modern communication systems including wireless sensor network applications entails the need to develop energy-efficient transmission techniques, especially dedicated to large, low-power networks (LPWAN) [7,8,9]. A promising approach seems to be the technique using the linear chirp spread spectrum (CSS) [10].

Time-frequency methods are widely used in the analysis, detection and parameter estimation of the LFM signals. The idea behind time-frequency processing based on time-frequency rate processing used in detection and the parameter estimation of LMF signals has been presented by authors in [11]. The promising time-frequency rate processing for LFM signals has been creatively developed and analysed in detail in the presented paper.

## 2. Problem Statement

The optimal approach to analysis of single-component LFM signals is based on the maximum likelihood estimator (MLE) [12,13,14]. However MLE is ineffective for the higher order polynomial phase signals, due to necessity of solving multidimensional optimisation problem with inevitable high numerical burden.

LFM signals embedded in noise can be described as follows:

(1) $$z_{r}\left(n\right)=z_{s}\left(n\right)+z_{w}\left(n\right)=b_{0}e^{j(a_{0}+a_{1}n+a_{2}n^{2})}+z_{w}\left(n\right),\;-\frac{N-1}{2}\leq n\leq \frac{N-1}{2},$$

where $z_{s}\left(n\right)$ is a noiseless polynomial phase signals with parameters $b_{0},a_{0},a_{1},a_{2}$ and $z_{w}\left(n\right)$ is complex white Gaussian noise of power $\sigma^{2}$.

The use of MLE approach for the signal $z_{r}\left(n\right)$ described by (1) leads to a two stage estimation algorithm where parameters $a_{1}$, $a_{2}$ ($a_{2}$ is also called CR parameter) are calculated in the following way:

(2) $$\left({\hat{a}}_{1},{\hat{a}}_{2}\right)=arg\max_{\left(a_{1},a_{2}\right)}\sum_{n=-\frac{N-1}{2}}^{\frac{N-1}{2}}z_{r}\left(n\right)e^{-j(a_{1}n+a_{2}n^{2})}.$$

Next, using ${\hat{a}}_{1},{\hat{a}}_{2}$ in the dechirping process, two remaining parameters $b_{0},a_{0}$ of the signal can be calculated as follows:

(3) $${\hat{b}}_{0}=\frac{1}{N}\sum_{n=-\frac{N-1}{2}}^{\frac{N-1}{2}}z_{r}\left(n\right)e^{-j({\hat{a}}_{1}n+{\hat{a}}_{2}n^{2})},$$

(4) $${\hat{a}}_{0}=angle\left(\sum_{n=-\frac{N-1}{2}}^{\frac{N-1}{2}}z_{r}\left(n\right)e^{-j({\hat{a}}_{1}n+{\hat{a}}_{2}n^{2})}\right).$$

There are several suboptimal approaches employed to the problem of parameter estimation of LFM signals. Each of them suffers from method-specific drawbacks but also shows method-specific advantages. The first generation of time-frequency (T-F) methods was based on the high-order ambiguity function (HAF), product HAF (PHAF), integrated generalized ambiguity function (IGAF) [15]. The chirp-Fourier transform or subspace-based algorithms (e.g., MUSIC and ESPRIT) [16,17] due to the non-stationary properties of chirp signals and the high rank of covariance matrix are much less useful than the maximum likelihood (ML) method. A variety of different methods were recommended for the analysis and estimation of LFM signals based on T-F processing for example extended generalized chirp transform [18]. The WVD and the spectrogram as the square modulus of the STFT show ideally linear dependence of frequency on time for LFM signals in T-F plane. However the STFT transform can be also used in parameter estimation of higher order polynomial phase signal (PPS) with polynomial order P>2 [19]. Next, the problem of detection and estimation of LFM signals can be solved using image processing methods, so it is reduced to the detection of line in an image, which is an easy-solved problem in pattern recognition [20,21,22]. The Radon-Wigner transform (RWT), the Wigner Hough transform (WHT) and the Radon-ambiguity transform (RAT) detect LFM signals in a time-frequency image by incoherent energy integration of Wigner-Ville distribution or ambiguity function (AF) in the image. Unfortunately these methods are complex and time consuming compared to other known methods such as those based on the chirp-Fourier transform and the MLE [15,23,24]. The Quasi-maximum-likelihood estimator (QML) is an extension of the STFT. In this method, the optimal window is searched in the STFT. Next, the STFT for the optimal window is performed to obtain rough estimates of parameters (including the $a_{2}$ parameter) [25], which are used as initial conditions in the ML procedure. The concept of the complex phase of the STFT results in additional new structures of instantaneous $a_{2}$ estimators for LFM signals [26].

In this paper, LFM signals defined by the polynomial phase of the second order are estimated by methods based on the CPF, which are in time–frequency rate (T-FR) processing domain. Due to nonlinearity in the exponent of the CPF transformation, this distribution cannot be calculated exactly by the Fourier transform, so sub-band decomposition in frequency rate (further denoted as Ω) domain should be done to reduce a number of operations for the CPF calculation. Instantaneous frequency rate is one of arguments of the CPF distribution whereas instantaneous frequency is an argument of well-known quadratic time-frequency distributions like the spectrogram or the WVD in the T-F plane. Although both the IFR and the IF are denoted by the same symbol Ω in relevant distributions, they have completely different properties. In general, even for multi-component signal case the IF as the first derivative with respect to time of the phase function can be calculated by the differentiation of the STFT phase. However the IFR as the second derivative of the phase function cannot be calculated by the second derivative of the STFT phase for the same signal [27,28]. When a signal is the PPS of the second order (LFM signals), it is possible to estimate IFR i.e., $a_{2}$ in alternative way by analysing peaks of the CPF [29]. Having had the IFR estimator, all parameters of LFM signals can be estimated. Hybrid CPF extensions such as the CPF-HAF and the high order CPF-Wigner-Ville (HOCPF-WD) [30] are dedicated to higher order PPS estimation and the $a_{2}$ parameter is included in the estimated set of parameters. Another extension called the integrated CPF (ICPF) refers to LFM signals only and offers good properties of IFR estimation [29].

## 3. Extended Forms of the Standard CPF

Initially, the CPF was a distribution intended for parameter estimation of quadratic FM signals only, but this distribution can be effectively used for parameter estimation of linear FM signals. The CPF distribution is given as [25,31]:

(5) $$CPF_{z_{r}}\left(n,\Omega \right)=\sum_{m=0}^{\frac{N-1}{2}}z_{r}\left(n+m\right)z_{r}\left(n-m\right)e^{-j\Omega m^{2}},\;-\frac{N-1}{2}\leq n\leq \frac{N-1}{2},$$

where Ω is an argument related to the IFR of signals. The IFR is defined in the form:

(6) $$IFR\left(n\right)=\frac{d^{2}\phi \left(n\right)}{dn^{2}},$$

where ϕ(n) is a signal phase. IFR estimate at time moment *n* can be obtained as following [25,31]:

(7) $$\hat{IFR}\left(n\right)=arg\max_{\Omega }\left|CPF\left(n,\Omega \right)\right|,$$

so the estimate of the IFR is an argument, which maximises the magnitude of the CPF at each *n*.

When computing the CPF formula, the product $z_{r}\left(n+m\right)z_{r}\left(m-n\right)$ is calculated first and for LFM signals this operation results in deterministic ($z_{det}\left(n\right)$) and random ($z_{rand}\left(n\right)$) components:

(8) $$z_{r}\left(n+m\right)z_{r}\left(m-n\right)=z_{det}\left(n\right)+z_{rand}\left(n\right)=b_{0}^{2}e^{j2[\left(a_{0}+a_{1}n+a_{2}n^{2}\right)+a_{2}m^{2}]}+z_{rand}\left(n\right).$$

The expression $2(a_{0}+a_{1}n+a_{2}n^{2})$ is a constant for given *n* and represents the ‘initial phase’, whereas the $\left(2a_{2}\right)$ is a quadratic phase parameter with respect to *m* and corresponds exactly to the IFR of a LFM signal. For $\Omega =2a_{2}$, the CPF attains the maximum, for which the Ω value is the IFR allowing to estimate the chirp parameter $a_{2}$. Then the estimate of $a_{1}$ can be found using the Fourier transform F of the dechirped signal $z_{rd}\left(n\right)$.

(9) $$z_{rd}\left(n\right)=z_{r}\left(n\right)e^{-j{\hat{a}}_{2}n^{2}},$$

(10) $${\hat{a}}_{1}=\arg\max_{a_{1}}\left[abs\left(F\left(z_{rd}\left(n\right)\right)\right)\right].$$

Parameters ${\hat{a}}_{0}$ and ${\hat{b}}_{0}$ are estimated in the same way as in (3) and (4).

The goal of our research is to develop method, which allow to improve performance of the estimation procedure. The objective is detection of LFM signals described by the parameters $a_{2}$, $a_{1}$, $a_{0}$ from the noisy observations and estimation of the $a_{2}$ parameter, with the accuracy close to the CRLB and with the SNR threshold as low as possible, in low SNR conditions. To achieve the intention, we propose two novel distributions: the SCPF and the PCPF build as extended forms of the standard CPF distribution. We evaluate the use of the SCPF and the PCPF by preliminary theoretical analysis and extensive simulation testing. The proposed extensions rely on the combination (multiplying (PCPF) or adding (SPCF)) of several CPF slices. The term slice is defined as the cross-section of the CPF taken at different time instant *n* (as in (11) and (12)). This approach results in obtaining new time-frequency rate distributions with different properties than the properties of standard CPF.

(11) $$SCPF\left(\Omega \right)=\sum_{n=0}^{L}CPF\left(n,\Omega \right),$$

where the SCPF is the sum of the CPFs at different time positions *n*.

(12) $$PCPF\left(\Omega \right)=\prod_{n=0}^{L}CPF\left(n,\Omega \right),$$

where the PCPF is the product of the CPFs at different time positions *n*.

It is expected that the multiplication and summing operations will amplify auto-terms of a signal and suppress the noise component which would decrease the Signal-to-Noise threshold (SNR threshold), under which the estimation process exhibits a rapid deterioration of estimation accuracy. It can be easily noticed that the extended forms show the same location of a peak related to the $a_{2}$ parameter as in the CPF. The CPF-based estimator for $a_{2}$ estimation is statistically efficient, since its mean squared error (MSE) asymptotically approaches the Cramer–Rao lower bound (CRLB) at high SNR at n=0 [25]. It is anticipated that extended estimators SCPF and PCPF of the $a_{2}$ parameter will reveal good statistical properties at much lower SNR threshold values than the CPF-based estimator. The estimation process of the $a_{2}$ parameter can be reformulated as the detection of LFM signals with the set of parameters $a_{0},a_{1},a_{2}$ in an observed signal, therefore the procedure of searching IFR spectrum peaks, which exceed the detection threshold level is proposed in the detection stage.

## 4. Detection of LFM Signals with Using CPF-Based Detectors

The issue of detection of LFM signals can be solved with use of binary hypotheses testing with two hypothesis ($H_{0}$ and $H_{1}$) on absence or presence of a signal in noise background formulated as following:

(13) $$H_{0}:z_{r}=z_{w},$$

(14) $$H_{1}:z_{r}=z_{s}+z_{w}.$$

The CPF-based detection procedure compares the highest peak of test statistics the TCPF and TSCPF, TPCPF proposed by authors with the threshold $\gamma_{XCPF}$. The thresholds can be calculated based on Neyman-Pearson criterion with assumed probability of false alarm ($P_{FA}$).

(15) $$TCPF=\max_{\Omega }\left|CPF\left(n,\Omega \right)\right|{≷}_{H_{0}}^{H_{1}}\gamma_{CPF},$$

(16) $$TSCPF=\max_{\Omega }\left|\sum_{n}CPF\left(n,\Omega \right)\right|{≷}_{H_{0}}^{H_{1}}\gamma_{SCPF},$$

(17) $$TPCPF=\max_{\Omega }\left|\prod_{n}CPF\left(n,\Omega \right)\right|{≷}_{H_{0}}^{H_{1}}\gamma_{PCPF}.$$

Due to the fact that test statistics are non-linear, the Monte Carlo simulations are used to determine the probability of signal detection for a given $P_{FA}$. The proposed detection method of LFM signals is equivalent to estimation of their CR ($a_{2}$ parameter).

**Figure 1:**
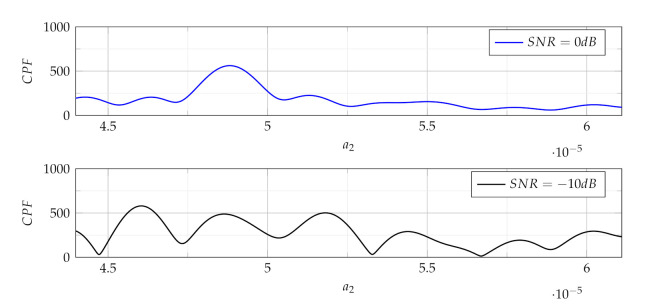
Examples of the CPF realisations used in the process of $a_{2}$ parameter estimation for two signals with different SNR values.

The concept of signal detection and estimation of its parameters using the CPF is shown in Figure 1, where two CPF realisations are presented for the up-chirp LFM signal with bandwidth $B_{chirp}=100$ kHz and parameters: $b_{0}=1$, $a_{0}=0$, $a_{1}=-0.05$, $a_{2}=4.8876\times 10^{-5}$. These parameters are also used in simulations presented in the Section 6. Two cases with SNR = 0 dB and SNR = −10 dB are shown. The position of Ω for which the CPF magnitude achieves the maximum value ($\Omega_{max}$) is searched. This position points out the estimated value of $a_{2}$ parameter as follows [29]:

(18) $${\hat{a}}_{2}=\frac{\Omega_{max}}{2}.$$

For a noiseless signal or signal with the high SNR (as can be seen in upper part of Figure 1), the maximum of the |CPF| clearly indicates the Ω value for which this maximum occurs. However for low SNR values, the maximum may be ambiguous as presented in lower part of Figure 1.

The flowchart of the $a_{2}$ parameter estimation can be summarised in the following way:

- calculate the CPF distribution (according to (5));
- calculate the SCPF or the PCPF distribution (according to (11) or (12));
- take the $\Omega_{max}$ value obtained from maximum value of the SCPF or the PCPF magnitude and calculate estimate of $a_{2}$ parameter (according to (18));
- moreover, $a_{1}$ estimate could be obtained in the classical way by searching maximum in Fourier spectrum after signal dechirping with use of ${\hat{a}}_{2}$, whereas $a_{0}$ and $b_{0}$ could be calculated according to (3) and (4).

## 5. Statistical Properties of the Chirp-Rate Parameter Estimators

In this section theoretical expression for the MSE of the parameter $a_{2}$ is developed. The exact formula for the MSE is difficult to derive because of high order of nonlinearities of the CPF-based estimator, so only the approximate formula is proposed. In the paper, the analysis of the MSE of the parameter $a_{2}$ is performed for the standard CPF-based estimator [31] and for two extended forms: the SCPF (first author’s idea) and the PCPF [32] as functions of SNR parameter.

The statistical analysis of the $a_{2}$ estimate is the most critical step because this parameter is used in the dechirping process and significantly affects the accuracy of the other parameter estimation. The CPF applied to a noiseless LFM signal ideally concentrates signal energy along the line $\Omega_{0}=2a_{2}$ for each time instant *n*, therefore $a_{2}$ parameter estimation can be calculated according to (18). Due to noise, the location of the maximum of the CPF for a noiseless LFM signal obtained for $\Omega_{0}$ may be moved by a random shift δΩ to a new location resulting in deviated estimate $\hat{\Omega }=\Omega_{0}+\delta \Omega$. The error δΩ of $a_{2}$ parameter estimation is evaluated by the MSE. Generally, the mean squared error comprise two components $MSE\left(\delta \Omega \right)=bias^{2}\left(\delta \Omega \right)+var\left(\delta \Omega \right)$, but IFR estimators for a phase polynomial signal are unbiased if $\frac{d^{k}\phi }{dn^{k}}=0$ for k>3. Therefore, the MSE is only the variance component $MSE\left(\delta \Omega \right)=E\{{\left(\delta \Omega \right)}^{2}\}$, where E{•} denotes the expectation operator. The derivation of the asymptotic MSE is based on the first order perturbation approach under assumption that the number of data points and SNRs tend to infinity [14].

Let’s assume that a function $g_{N}\left(\Omega \right)$ which depends on a real variable Ω and an integer *N* exists. The magnitude of that function $g_{N}\left(\Omega \right)$ has the same global maximum at $\Omega =\Omega_{0}$ as the magnitude of the CPF. The perturbation function $\delta g_{N}\left(\Omega \right)$ causes the small perturbation of the function $g_{N}\left(\Omega \right)$ because of noise. This perturbation will cause that the point $\Omega_{0}$ of global maximum of the $g_{N}\left(\Omega \right)$ is modified by an amount δΩ and the global maximum is shifted from $\Omega_{0}$ to $\Omega_{0}+\delta \Omega$.

A first order perturbation for perturbation δΩ is given by the relation [31]:

(19) $$\delta \Omega \approx -\frac{B}{A}\;,$$

where

(20) $$A=2Re\left\{g_{N}\left(\Omega_{0}\right){\frac{\partial^{2}g_{N}^{*}\left(\Omega_{0}\right)}{\partial \Omega }}^{2}+\frac{\partial g_{N}\left(\Omega_{0}\right)}{\partial \Omega }\frac{\partial^{2}g_{N}^{*}\left(\Omega_{0}\right)}{\partial \Omega }\right\},$$

and

(21) $$B=2Re\left\{g_{N}\left(\Omega_{0}\right)\frac{\partial \delta g_{N}^{*}\left(\Omega_{0}\right)}{\partial \Omega }+\frac{\partial g_{N}\left(\Omega_{0}\right)}{\partial \Omega }\delta g_{N}^{*}\left(\Omega_{0}\right)\right\}.$$

The mean squared error of δΩ is described by:

(22) $$E\left\{{\left(\delta \Omega \right)}^{2}\right\}\approx \frac{E\{B^{2}\}}{A^{2}}.$$

For the considered CPF-based estimation problem, the function $g_{N}\left(\Omega \right)$ can be defined in the following form [31]:

(23) $$g_{N}\left(\Omega \right)=CPF_{z_{s}}\left(n,\Omega \right).$$

The perturbation function $\delta g_{N}\left(\Omega \right)$ is assumed as:

(24) $$\delta g_{N}\left(\Omega \right)=\sum_{m=0}^{\left(N-1)/2-|n|\right)}z_{ws}\left(n,m\right)e^{-j\Omega m^{2}},$$

where

(25) $$z_{ws}\left(n,m\right)=z_{s}\left(n+m\right)z_{w}\left(n-m\right)+z_{s}\left(n-m\right)z_{w}\left(n+m\right)+z_{w}\left(n-m\right)z_{w}\left(n+m\right),$$

includes the interference of noise. To derive the perturbation δΩ equations (20) and (21) should be used. The approximate relation below shows the MSE for given SNR (i.e., $b_{0}^{2}/\sigma^{2}$) and *N* for the slice at n=0 [31]:

(26) $$E\left\{{\left(\delta \Omega \right)}^{2}\right\}\approx \frac{360\left(1+\frac{1}{2SNR}\right)}{N^{5}SNR}.$$

For n≠0 approximate properties are changed due to reduced number of samples of the kernel $z_{r}\left(n+m\right)z_{r}\left(n-m\right)$. The variance of the δΩ estimator for any *n* is given by the approximate relation [31]:

(27) $$E\left\{{\left(\delta \Omega \right)}^{2}\right\}\approx \frac{45}{4{\left(\frac{N}{2}-\left|n\right|\right)}^{5}SNR}.$$

The perturbation δΩ can be expressed by the perturbation $\delta a_{2}$ according to the relation $\Omega =2a_{2}$. For the slice n=0 the variance of the $\delta a_{2}$ can be expressed as [31]:

(28) $$E\left\{{\left(\delta a_{2}\right)}^{2}\right\}\approx \frac{90\left(1+\frac{1}{2SNR}\right)}{N^{5}SNR}=\left(1+\frac{1}{2SNR}\right)CRLB\left\{{\hat{a}}_{2}\right\},$$

where the CRLB of the parameter $a_{2}$ [14] is approximately determined by:

(29) $$CRLB\left\{{\hat{a}}_{2}\right\}=\frac{90}{N^{5}SNR}.$$

The combination of different forms of the standard CPF is a promising idea to improve performance of the estimation and detection tasks. Multiplication and summing of *L* slices would be more effective because of different impact of noise on the modified estimators. Noise can add destructively while the signal can add constructively, that’s why noise may have relatively smaller influence on the perturbation δΩ. Moreover both extended estimators the PCPF and the SCPF for noiseless case achieve a maximum at $\Omega =2a_{2}$. Examining the relation (27) it can be seen that the vital statistical properties lie in the vicinity of the slice n=0, therefore a few L<<N slices are sufficiently informative.

### 5.1. Analysis of Statistical Properties of SCPF-Based Detector

The goal of the SCPF-based estimator is improvement of statistical properties of estimators and reduction of the SNR threshold. To derive the MSE of the SCPF for the first two slices based on the first order perturbation, two functions $g_{N}\left(\Omega \right)$ and $\delta g_{N}\left(\Omega \right)$ are required, which take the form proposed by authors:

(30) $$g_{N}\left(\Omega \right)=\sum_{n=0}^{1}\sum_{m=0}^{(N-1)/2}s_{1}s_{2}e^{-j\Omega m^{2}},$$

(31) $$\delta g_{N}\left(\Omega \right)=\sum_{n=0}^{1}\sum_{m=0}^{\left(N-1)/2-|n\right|}z_{ws}\left(n,m\right)e^{-j\Omega m^{2}},$$

where

$$s_{1}=z_{s}\left(n+m\right),\;s_{2}=z_{s}\left(n-m\right),$$

$$z_{ws}=\left(s_{1}+z_{w1}\right)\left(s_{2}+z_{w2}\right)=s_{1}s_{2}+s_{1}z_{w2}+s_{2}z_{w1}+z_{w1}z_{w2}.$$

For further notation simplicity the notation $w_{1}=z_{w1}$ and $w_{2}=z_{w2}$ is taken. The mean squared value of ${\left(\delta \Omega \right)}^{2}$ can be expressed in terms of *A* and *B* according to the Formula (22). For the considered by authors scenario, the term *A* is calculated by the relation:

(32) $$A=\frac{\partial^{2}g_{N}\left(\Omega_{0}\right)}{\partial \Omega^{2}}=-b_{0}^{2}\frac{3N^{5}-10N^{3}+7N}{240}.$$

Expressions for *N* slices for *B* and its conjugate $B^{*}$ according to (21) are shown in (33) and (34) respectively.

(33) $$\begin{array}{cc} B & =\sum_{n=0}^{N-1}\sum_{m=0}^{\left(N-1)/2-|n\right|}m^{2}z_{ws}\left(n,m\right)e^{-j(\Omega m^{2}-\frac{\pi }{2})}=\\ \; & =\sum_{n=0}^{N-1}\sum_{m=0}^{\left(N-1)/2-|n\right|}m^{2}\left(s_{1}s_{2}+s_{1}w_{2}+s_{2}w_{1}+w_{1}w_{2}\right)e^{-j(\Omega m^{2}-\frac{\pi }{2})} \end{array}$$

(34) $$\begin{array}{cc} B^{*} & =\sum_{k=0}^{N-1}\sum_{l=0}^{\left(N-1)/2-|n\right|}l^{2}z_{ws}^{*}\left(k,l\right)e^{-j(\Omega l^{2}-\frac{\pi }{2})}=\\ \; & =\sum_{k=0}^{N-1}\sum_{l=0}^{\left(N-1)/2-|n\right|}l^{2}\left(s_{3}^{*}s_{4}^{*}+s_{3}^{*}w_{4}^{*}+s_{4}^{*}w_{3}^{*}+w_{3}^{*}w_{4}^{*}\right)e^{-j(\Omega l^{2}-\frac{\pi }{2})} \end{array}$$

The intermediate step for $E\{BB^{*}\}$ is derived in (35) and (36):

(35) $$\begin{array}{cc} E\{BB^{*}\}= & \sum_{n=0}^{N-1}\sum_{m=0}^{(N-1)/2}m^{2}\left(s_{1}s_{2}+s_{1}w_{2}+s_{2}w_{1}+w_{1}w_{2}\right)e^{-j(\Omega m^{2}-\frac{\pi }{2})}\\ \; & \sum_{k=0}^{N-1}\sum_{l=0}^{(N-1)/2}l^{2}\left(s_{3}^{*}s_{4}^{*}+s_{3}^{*}w_{4}^{*}+s_{4}^{*}w_{3}^{*}s+w_{3}^{*}w_{4}^{*}\right)e^{-j(\Omega l^{2}-\frac{\pi }{2})}=\\ = & \sum_{n=0}^{N-1}\sum_{m=0}^{(N-1)/2}\sum_{k=0}^{N-1}\sum_{l=0}^{(N-1)/2}m^{2}l^{2}\left(s_{1}s_{2}+s_{1}w_{2}+s_{2}w_{1}+w_{1}w_{2}\right)e^{-j(\Omega m^{2}-\frac{\pi }{2})}\\ \; & \left(s_{3}^{*}s_{4}^{*}+s_{3}^{*}w_{4}^{*}+s_{4}^{*}w_{3}^{*}+w_{3}^{*}w_{4}^{*}\right)e^{-j(\Omega l^{2}-\frac{\pi }{2})} \end{array}$$

Based on the high-order moment properties of the Gaussian random variable [33], $E\{BB^{*}\}$ can be computed as multiple summations of the Kronecker delta functions.

(36) $$\begin{array}{cc} E\{BB^{*}\}= & b_{0}^{4}\sum_{m=0}^{(N-1)/2}\sum_{l=0}^{(N-1)/2}m^{2}l^{2}+\sum_{n=0}^{N-1}\sum_{m=0}^{(N-1)/2}\sum_{k=0}^{N-1}\sum_{l=0}^{(N-1)/2}m^{2}l^{2}\\ \; & \{b_{0}^{2}\sigma^{2}(\delta \left(n-k-m+l\right)+\delta \left(n-k-m-l\right)+\\ \; & +\delta (n-k+m+l)+\delta (n-k+m-l))+\\ \; & +\sigma^{4}\delta \left(n-k+m-l\right)\delta \left(n-k-m+l\right)+\\ \; & +\sigma^{4}\delta \left(n-k+m-l\right)\delta \left(n-k-m+l\right)\} \end{array}$$

Since Kronecker’s deltas δ(n−k−m−l) and δ(n−k+m+l) never yields one, these deltas are removed from further derivation of formulas. In the next step of deriving the relation $E\{BB^{*}\}$ the number of slices was limited to two in the SPCF-based estimator. Omitting the effect of the signal itself and taking only impact of noise, the sum of two slices for n=0:1, k=0:1 leads to the final formula:

(37) $$\begin{array}{cc} E\{\left(\delta \Omega^{2}\right)\}\approx & E\left\{\frac{BB^{*}}{A^{2}}\right\}=\{b_{0}^{2}\sigma^{2}8\left(\frac{N-1}{2}\right)\left(\frac{N-1}{2}+1\right)\left(2\frac{N-1}{2}+1\right)\\ \; & \left(3{\left(\frac{N-1}{2}\right)}^{2}+3\frac{N-1}{2}-1\right)\frac{1}{30}-b_{0}^{2}\sigma^{2}8\left(\frac{{\left(\frac{N-1}{2}\right)}^{2}+\frac{N-1}{2}}{2}\right)+\\ \; & +4b_{0}^{2}\sigma^{2}\left(\frac{N-1}{2}\right)\left(\frac{N-1}{2}+1\right)\left(2\frac{N-1}{2}+1\right)\frac{1}{6}+\\ \; & +\sigma^{2}2\left(\frac{N-1}{2}\right)\left(\frac{N-1}{2}+1\right)\left(2\frac{N-1}{2}+1\right)\\ \; & \left(3{\left(\frac{N-1}{2}\right)}^{2}+3\frac{N-1}{2}-1\right)\}\frac{1}{30}\frac{1}{{\left(-b_{0}^{2}\frac{3N^{5}-10N^{3}+7N}{240}\right)}^{2}} \end{array}$$

It is assumed that the estimate ${\hat{a}}_{2}$ differ from true value of $a_{2}$ by $\delta a_{2}$. Taking the relation $\Omega =2a_{2}$ and (22) into account, the $E\{{\left(\delta a_{2}\right)}^{2}\}$ is expressed in the final form developed by authors:

(38) $$\begin{array}{c} E\left\{{\left(\delta a_{2}\right)}^{2}\right\}\approx \frac{60\left(6SNR\left(\left(2N-5\right)N^{2}+2N+5\right)+N\left(3N^{2}-7\right)\right)}{N^{2}SNR^{2}b_{0}^{4}\left(N^{2}-1\right){\left(3N^{2}-7\right)}^{2}}. \end{array}$$

**Figure 2:**
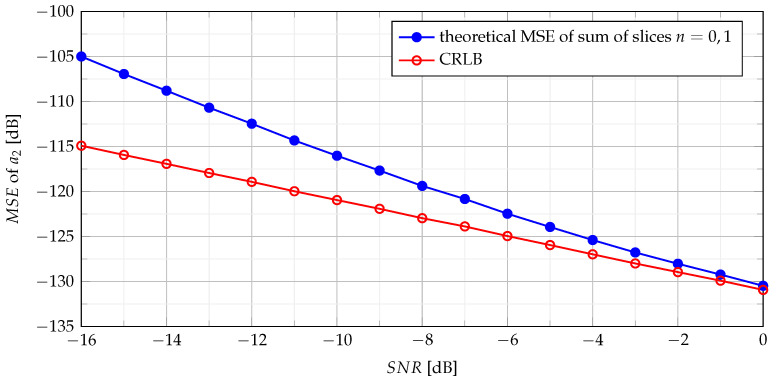
Theoretical MSE of sum of slices n={0,1}.

The relation of the theoretical MSE (38) and the CRLB (29) versus the SNR is presented in Figure 2.

The proposed approach is focused only on the assessment of the $a_{2}$ parameter. After the estimate of the parameter $a_{2}$ is obtained, the signal $z_{r}$ is dechirped and the final signal is the linear phase signal in additive noise, therefore conventional estimation methods can be used for remaining parameters.

### 5.2. Analysis of Statistical Properties of PCPF-Based Detector

The theoretical analysis of statistical properties of the product of slices at different time positions in the PCPF is limited to product of any two slices at $n=n_{1}$ and $n=n_{2}$. Derivation of approximate expressions using the first order perturbation for the mean squared errors of the estimated parameter $a_{2}$ for higher number of slices of extended estimators requires very high computational load [32]. Therefore estimation results for higher number of slices are verified only by numerical simulations.

The function $g_{N}\left(\Omega \right)$ and the function $\delta g_{N}\left(\Omega \right)$ in the PCPF case are defined as follows [32]:

(39) $$g_{N}\left(\Omega \right)=\sum_{m=0}^{N_{1}}z_{s}\left(n_{1}+m\right)z_{s}\left(n_{1}-m\right)e^{-j\Omega m^{2}}\cdot \sum_{m=0}^{N_{2}}z_{s}\left(n_{2}+m\right)z_{s}\left(n_{2}-m\right)e^{-j\Omega m^{2}}$$

(40) $$\begin{array}{cc} \delta g_{N}\left(\Omega \right) & =\sum_{m=0}^{N_{1}}z_{s}\left(n_{1}+m\right)z_{s}\left(n_{1}-m\right)e^{-j\Omega m^{2}}\sum_{m=0}^{N_{2}}z_{ws}\left(n_{2}\right)e^{-j\Omega m^{2}}+\\ \; & +\sum_{m=0}^{N_{2}}z_{s}\left(n_{2}+m\right)z_{s}\left(n_{2}-m\right)e^{-j\Omega m^{2}}\sum_{m=0}^{N_{1}}z_{ws}\left(n_{1}\right)e^{-j\Omega m^{2}} \end{array}$$

where

(41) $$z_{ws}\left(n_{1}\right)=z_{s}\left(n_{1}+m\right)z_{w}\left(n_{1}-m\right)+z_{s}\left(n_{1}-m\right)z_{w}\left(n_{1}+m\right)+z_{w}\left(n_{1}-m\right)z_{w}\left(n_{1}+m\right),$$

(42) $$z_{ws}\left(n_{2}\right)=z_{s}\left(n_{2}+m\right)z_{w}\left(n_{2}-m\right)+z_{s}\left(n_{2}-m\right)z_{w}\left(n_{2}+m\right)+z_{w}\left(n_{2}-m\right)z_{w}\left(n_{2}+m\right).$$

**Figure 3:**
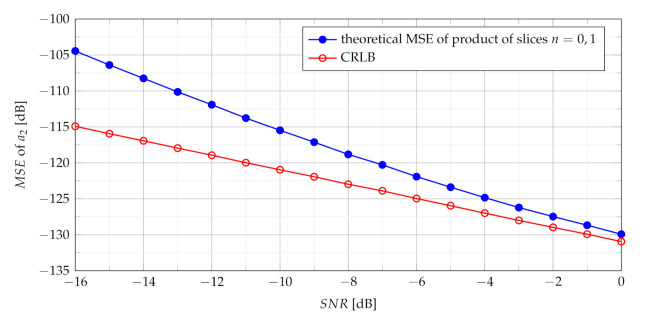
Theoretical MSE of product of slices n={0,1}.

As previously, $g_{N}\left(\Omega \right)$ contains only signal-related terms and therefore is deterministic, whereas $\delta g_{N}\left(\Omega \right)$ includes interacting signal-and-noise terms which are random. Functions found in Equations (20) and (21) may be approximated by the relation [32]:

(43) $$g_{N}\left(\Omega_{0}\right)\approx b_{0}^{2}K^{2}N_{1}N_{2},$$

(44) $$\frac{\partial g_{N}\left(\Omega_{0}\right)}{\delta \Omega }\approx -jb_{0}^{4}K^{2}\frac{1}{3}\left(N_{1}N_{2}^{3}+N_{1}^{3}N_{2}\right),$$

(45) $$\frac{\partial^{2}g_{N}\left(\Omega_{0}\right)}{\delta \Omega^{2}}\approx -b_{0}^{4}K^{2}\frac{1}{45}\left(9N_{1}N_{2}^{5}+10N_{1}^{3}N_{2}^{3}+9N_{1}^{5}N_{2}\right),$$

(46) $$\delta g_{N}\left(\Omega_{0}\right)\approx b_{0}^{2}KN_{2}\sum_{m=0}^{N_{1}}z_{ws}\left(n_{1}\right)e^{-j\Omega_{0}m^{2}}+b_{0}^{2}KN_{1}\sum_{m=0}^{N_{2}}z_{ws}\left(n_{2}\right)e^{-j\Omega_{0}m^{2}},$$

(47) $$\begin{array}{cc} \frac{\partial \delta g_{N}\left(\Omega_{0}\right)}{\delta \Omega } & \approx jb_{0}^{2}KN_{2}\sum_{m=0}^{N_{1}}\left(m^{2}+\frac{N_{2}^{2}}{3}\right)z_{ws}\left(n_{1}\right)e^{-j\Omega_{0}m^{2}}-\\ \; & -jb_{0}^{2}KN_{1}\sum_{m=0}^{N_{2}}\left(m^{2}+\frac{N_{1}^{2}}{3}\right)z_{ws}\left(n_{2}\right)e^{-j\Omega_{0}m^{2}} \end{array}$$

where $\Omega_{0}$ is that value when $\Omega =2a_{2}$, $N_{1}=\left(N-1\right)/2-n_{1}$, $N_{2}=\left(N-1\right)/2-n_{2}$ and $K=e^{j(2a_{0}+a_{1}\left(n_{1}+n_{2}\right)+a_{2}{\left(n_{1}+n_{2}\right)}^{2})}$. Inserting (43)–(47) into (20) and (21), the MSE of the $\delta a_{2}$ can be approximately given by the relation [32]:

(48) $$\begin{array}{cc} E\left\{{\left(\delta a_{2}\right)}^{2}\right\} & \approx \frac{45\left[\left(46N_{1}^{5}N_{2}^{2}+16N_{1}^{2}N_{2}^{5}-14N_{1}^{6}N_{2}\right)+\frac{8N_{1}^{5}N_{2}^{2}+8N_{1}^{2}N_{2}^{5}}{SNR}\right]}{256SNR{\left(N_{1}N_{2}^{5}+N_{1}^{5}N_{2}\right)}^{2}}+\\ \; & +\frac{45(32N_{1}^{6}N_{2}-30N_{1}^{2}N_{2}^{5}+14N_{1}N_{2}^{6})}{256SNR{\left(N_{1}N_{2}^{5}+N_{1}^{5}N_{2}\right)}^{2}}1\left(N-4n_{1}\right) \end{array}$$

where 1(·) is the unit step function. General expression (48) is adopted for the analysis of the product of the first two slices $n_{1}=0$ and $n_{2}=1$ due to their important role in building of the MSE formula. As previously mentioned, the estimate ${\hat{a}}_{2}$ differs from true value of $a_{2}$ by $\delta a_{2}$. The relation of the theoretical MSE (48) and CRLB (29) versus SNR is presented in Figure 3.

## 6. Simulations Results

Simulation investigations of the proposed algorithms were carried out for up-chirp LFM waveform. The parameters of signal modelled as in (1) were: $b_{0}=1$, $a_{0}=0$, $a_{1}=\left(-B_{chirp}T_{s}\right)/2$, $a_{2}=\left(B_{chirp}T_{s}\right)/\left(2N\right)$. We assumed the bandwidth of the LFM signal $B_{chirp}=100$ kHz and time duration of the signal equal to $NT_{s}$ where N=1023 is the number of samples taken every sampling period $T_{s}=10^{-6}s$. The noise $z_{w}\left(n\right)$ was assumed to be complex Gaussian with variance depending on SNR. To assess the proposed detection methods based on test statistics: TCPF (15), TSCPF (16) and TPCPF (17), Monte-Carlo simulations for $N_{s}=10^{4}$ runs were carried out. The probability of detection ($P_{D}$) for thresholds established for assumed probabilities of false alarm ($P_{FA}$) in various SNR conditions has been determined.

**Figure 4:**
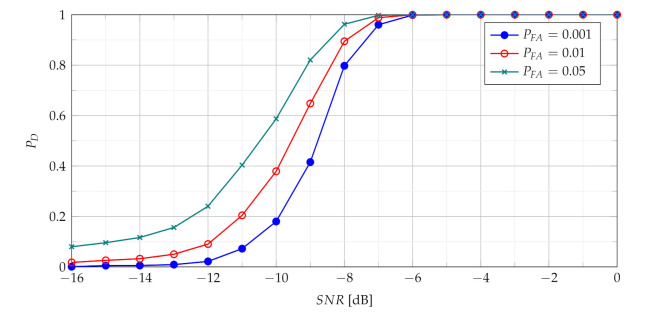
Probability of detection with use of the TCPF statistics for different $P_{FA}$ in various SNR conditions.

The Figure 4 presents probability of detection with use of the TCPF statistics for different $P_{FA}$ in various SNR conditions.

**Figure 5:**
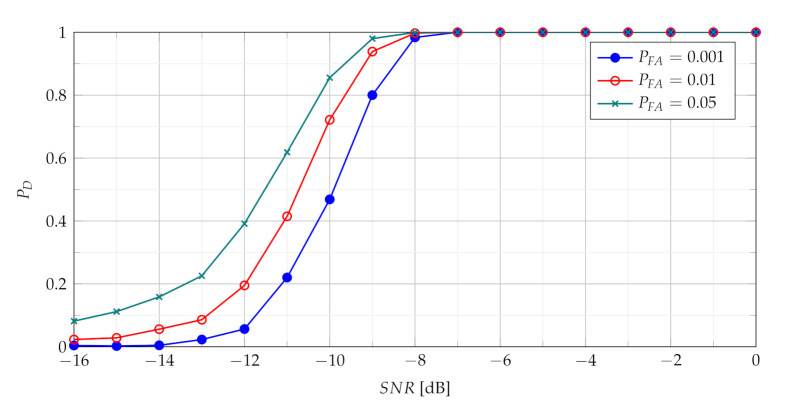
Probability of detection with use of the TSCPF statistics employing slices n={0,1}.

**Figure 6:**
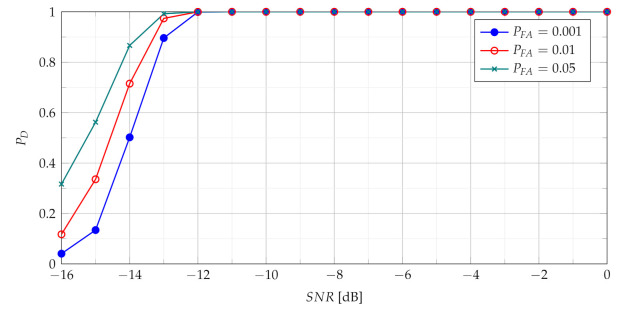
Probability of detection with use of the TSCPF statistics employing slices n={0,1,⋯,30}.

**Figure 7:**
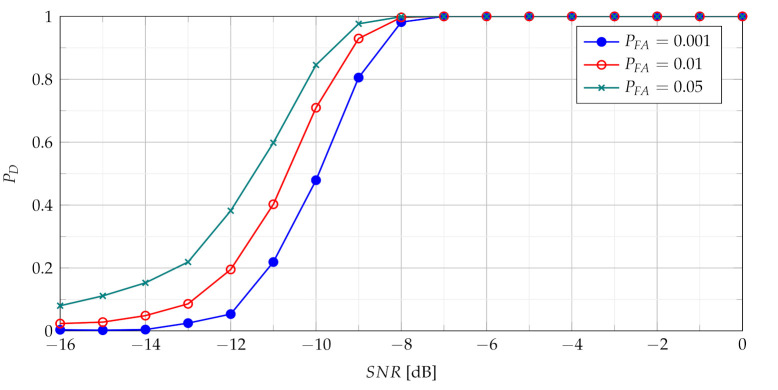
Probability of detection with use of the TPCPF statistics employing slices n={0,1}.

**Figure 8:**
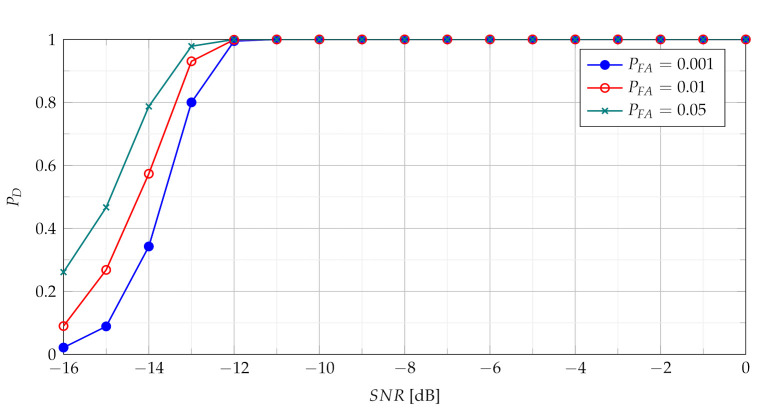
Probability of detection with use of the TPCPF statistics employing slices n={0,1,⋯,30}.

The $P_{D}$ as function of SNR presented in Figure 4 characterises the performance of the basic detector based on TCPF statistics. It may be treated as basis to assess the effectiveness of the more advanced detectors based on TSCPF and TPCPF, which $P_{D}$ versus SNR for various $P_{FA}$ are shown in Figure 5, Figure 6, Figure 7 and Figure 8.

In Figure 5 and Figure 7 TSCPF and TPCPF statistics which employ only first two slices (n={0,1}) are presented while the scenario with first thirty one slices (n={0,1,⋯,30}) is considered in Figure 6 and Figure 8.

Comparing Figure 5 with Figure 4 leads to observation that the TSCPF statistics with only two slices significantly increases detection performance in relation to the standard TCPF. Moreover Figure 5 and Figure 6 allow to notice considerable increase of $P_{D}$ with growing number of slices, however on the cost of rising computational burden.

**Figure 9:**
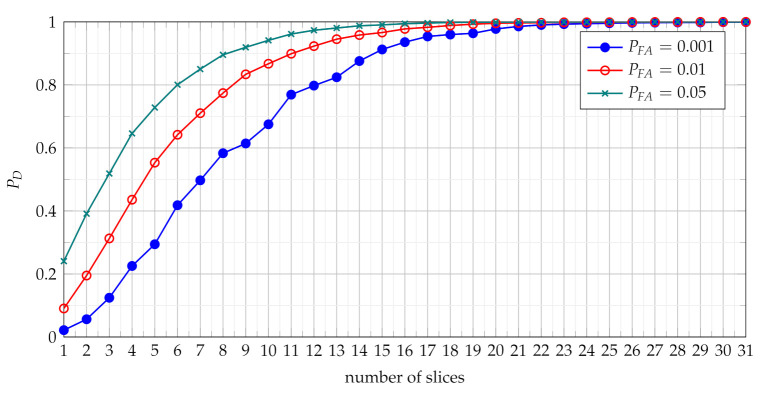
Dependence of the probability of detection on number of slices used in the TSCPF statistics for different $P_{FA}$ and SNR=−12 dB.

**Figure 10:**
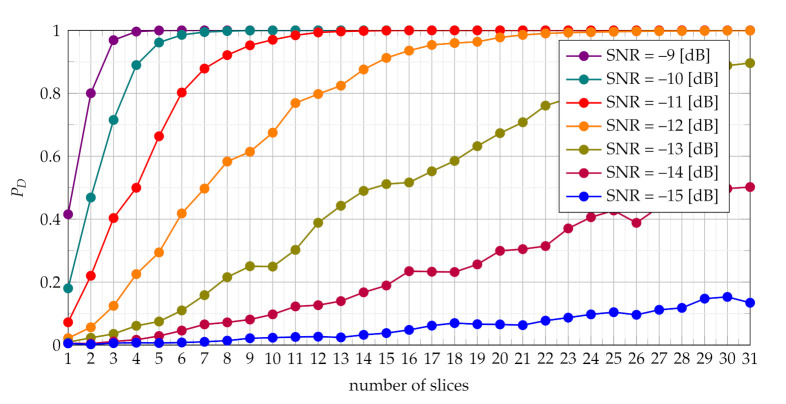
Dependence of the probability of detection on number of slices used in the TSCPF statistics for different SNR and $P_{FA}=0.001$.

Comparisons of characteristics shown in Figure 5, Figure 6, Figure 7 and Figure 8 lead to conclusion that the efficiency of TSCPF and TPCPF statistics is very similar. Moreover it can be clearly seen that detection performance increases evidently with growing number of slices for both the TSCPF and the TPCPF. The investigation results on this issue is presented in Figure 9 and Figure 10, where the relation between $P_{D}$ and number of slices for different SNR and $P_{FA}$ are analysed.

The results shown in Figure 9 and Figure 10 lead to the conclusion that a certain balance between the performance and computational load should be considered in particular cases.

**Figure 11:**
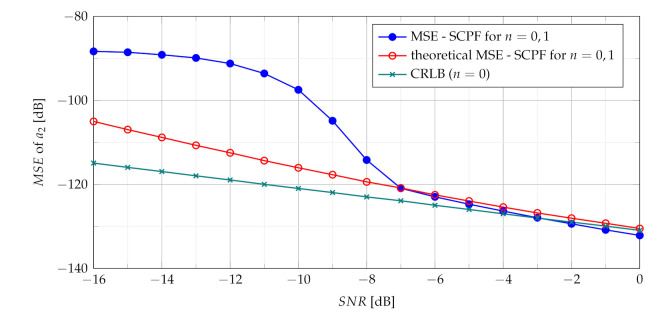
Theoretical and measured MSEs of the SCPF for n={0,1}.

The assessment of the CR estimate accuracy has been performed using SCPF and PCPF estimators. Numerical examples verify the theoretical results and show that the proposed SCPF and PCPF estimators outperform the standard CPF-based estimator. Figure 11 shows the theoretical and simulation results of the MSE of the CR parameter using the SCPF-based estimator.

**Figure 12:**
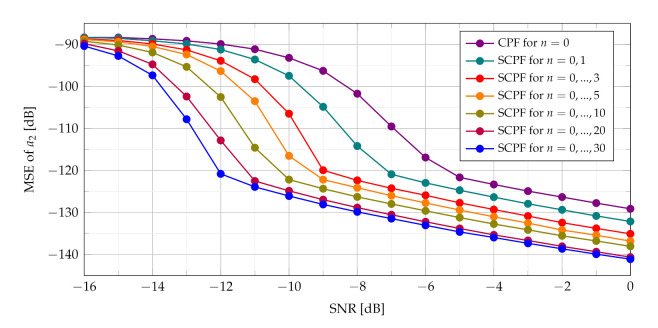
Influence of the number of SCPF slices on MSE.

**Figure 13:**
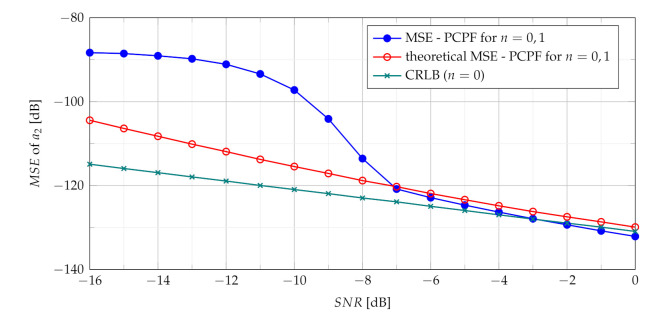
Theoretical and measured MSEs of the PCPF for n={0,1}.

**Figure 14:**
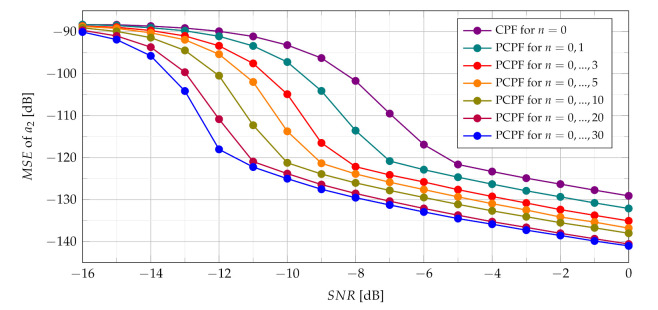
Influence of the number of PCPF slices on MSE.

In presence of heavy noise the maximum of the SCPF may occur at the true value of the $a_{2}$ or may occur away from this true value. This last scenario is known as the ‘outlier’ scenario, which may initialize the SNR threshold phenomenon clearly visible in MSE characteristics presented in Figure 11. The SNR threshold effects arise when the probability of an outlier becomes significant. The SNR threshold can be defined as a level of SNR at which the accuracy of estimation deteriorates rapidly and significantly differs from the theoretical MSE. The SNR threshold effect for various number of slices is presented in Figure 12. The explanation of the phenomenon can be also given by comparing the detection characteristics presented in Figure 5, Figure 6, Figure 7 and Figure 8 with the MSE characteristics presented in Figure 11, Figure 12, Figure 13 and Figure 14. As can be seen the SNR threshold occurs when the probability of detection decreases below 1. It means that some of the CR estimates are calculated with growing errors which lead to rapid increase of the MSE.

As can be seen in Figure 12 increasing the number of slices significantly changes the SNR threshold. With an increase of the number of slices in the SCPF, noise influence is reduced resulting in the lowering of the SNR threshold.

The PCPF algorithm for the same parameters as in the SCPF algorithm is applied and results of experiments are presented in Figure 13 and Figure 14. The SNR threshold phenomenon is also visible in obtained MSEs.

It is worth noting that for the SNR above the SNR threshold the simulation results approach closely the theoretical result (as presented in Figure 11 and Figure 13).

The simulations results presented in Figure 14 demonstrate that SNR threshold is substantially smaller for product of bigger number of slices compared to the standard CPF estimator and product of slices n={0,1} in the PCPF.

**Figure 15:**
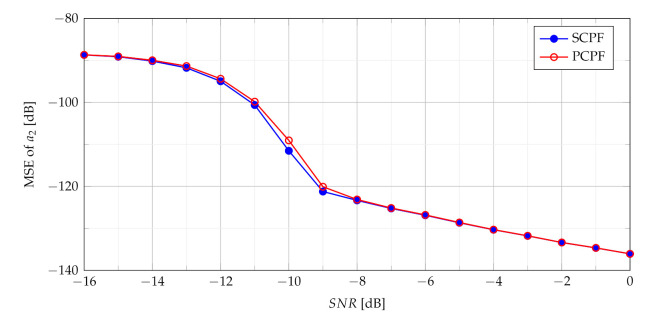
MSE of the SCPF-based estimator and the PCPF-based estimator for n={0,⋯,4} slices.

It can be concluded that expected lowering of the SNR threshold is achieved by summing and multiplying of the CPF slices, which is a novel approach proposed in the paper. As shown in Figure 15 despite different calculations of both the SPCF and the PCPF obtained MSEs demonstrate similar results.

As outlined in Section 2, there are a number of methods presented in literature which are dedicated to estimation of the parameters of the PPS. The estimation algorithms can be divided into three groups:

- maximum likelihood estimators,
- estimation methods in T-F plane,
- estimation methods in T-FR plane.

Simulations results presented in literature can be used as a basis for comparison, however most of them were performed on shorter lengths of signals, e.g., N=64, N=128, N=256. Simulation research examined in the paper were carried out for number of samples N=1023, which resulted from the assumed frequency bandwidth $B=100\times 10^{3}$ [Hz] of the LFM signal and the sampling frequency $fs=1\times 10^{6}$ [Hz], which corresponded to the actual LFM signals. In order to compare the performance of algorithms presented in the paper with results reported in the literature, experiments were repeated with new experimental parameters N=255 and N=63 with adequate lowering of sampling rate (e.g., $fs=255/1023\times 10^{6}$ for N=255), which ensures the same value of *B*, $a_{0}$, $a_{1}$, $a_{2}$ as in the main experiment.

**Figure 16:**
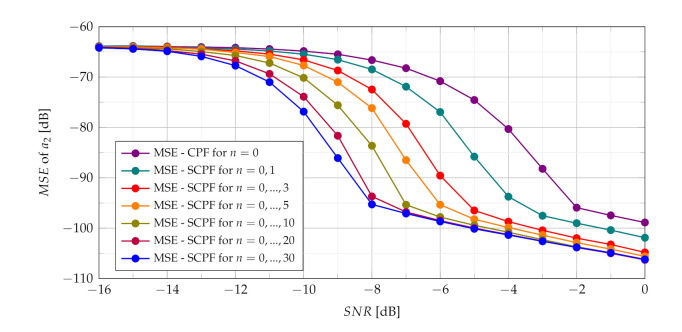
Influence of the number of SCPF slices on MSE for N=255.

**Figure 17:**
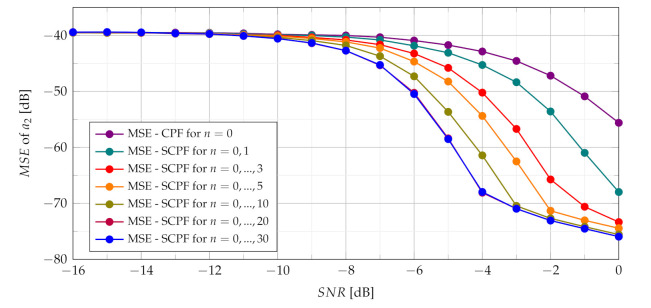
Influence of the number of SCPF slices on MSE for N=63.

Presented results of estimation characteristics (MSE) for shorter signals are shown in Figure 16 and Figure 17 for the SCPF-based estimator. Results for the PCPF-based estimator are similar as those in Figure 16 and Figure 17.

The estimation results presented in Figure 16 and Figure 17 reveal the SNR threshold th=−8 dB and th=−4 dB for N=255 and N=63 respectively, both with 31 slices. Comparison of these results with results for longer signal (N=1023) shown in Figure 12 points out loss by 4 dB and 8 dB in location of the SNR threshold. The results obtained with use of the proposed method can be compared with estimation results with use of the ICPF-based estimator from [29], where the SNR threshold was th=−8 dB for N=64 but with bigger number (64) of slices.

**Figure 18:**
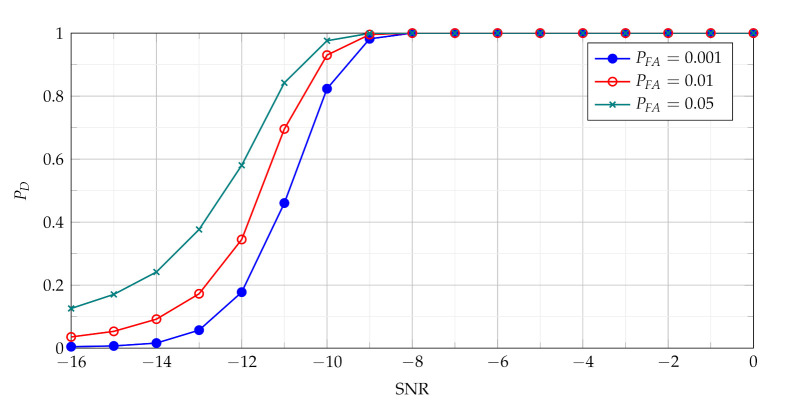
Probability of detection with use of the TSCPF statistics employing slices n={0,⋯,30} for N=255.

**Figure 19:**
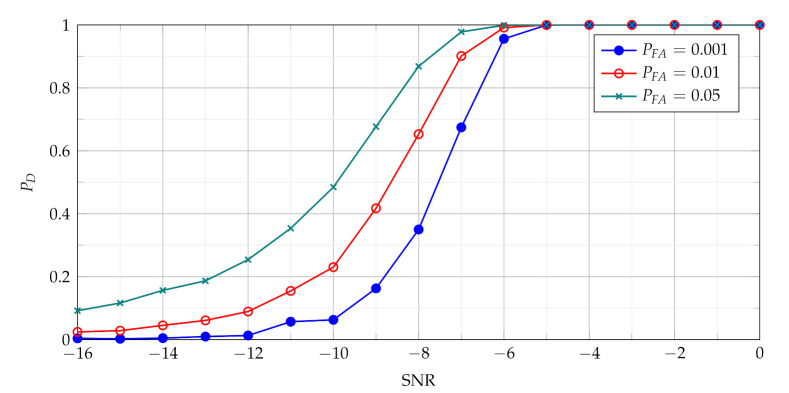
Probability of detection with use of the TSCPF statistics employing slices n={0,⋯,30} for N=63.

The detection algorithms considered in the paper are formulated as searching of $a_{2}$ parameter in the T-FR plane. The detection characteristics with N=255 and N=63 based on the TSCPF statistics used for detection are presented in Figure 18 and Figure 19. Results for the TPCPF statistics are similar. The results for TSCPF statistics and N=63 can be directly compared with the detection characteristics using the CPF-based detector and the ICPF-based detector presented in [29].

The comparison of results presented in Figure 19 for $P_{FA}=0.01$ and results presented in [29] indicates that detector based on the TSCPF statistics outperforms detector based on the TICPF statistics. It should be noticed that the detection algorithm from [29] uses 64 slices whereas the algorithm proposed in the paper only 31 slices.

Moreover, as can be seen in Figure 6, Figure 10, Figure 18 and Figure 19 detection achieves better efficiency with increasing number of signal samples and with increasing number of slices.

The extended comparison of the obtained results with results from the literature, which apply both selected time-frequency (T-F) methods and selected time-frequency rate (T-FR), is presented in the Table 1. The SNR threshold was selected as an evaluation method.

As can be seen in the Table 1, the proposed detection and estimation methods show the significantly lower SNR threshold than other algorithms used for detection and estimation of LFM signals.

## 7. Conclusions

This paper has presented the new approach for both detection and parameter estimation of LFM signals based on time-frequency rate distributions with use of developed SCPF and PCPF hybrid structures. It should be noted that most papers refer to well-known processing in the T-F plane, whereas the processing in T-FR isn’t well examined yet, so the paper is part of efforts to explore this area. The advantages of the developed approach result from the simplicity of the calculation of the SCPF-based estimator and PCPF–based estimator due to optimisation only in 1-D space. The obtained analytical results and performed simulations have shown effectiveness of the proposed methods. It has been proved, that the developed SCPF and PCPF as extended forms of the standard CPF reveal noticeable increase in the detection and estimation efficiency. Statistical properties of chirp-rate parameter estimation achieved with the PCPF-based and SCPF-based estimators turned out to be more accurate compared to methods based on the standard CPF or other combinations of distributions known in the literature. PCPF-based and SCPF-based T-FR estimators are also characterised by lower order of nonlinearities, which results from preserving bilinear transformation in the proposed hybrids of the CPF. It results in a lower SNR threshold compared to other time-frequency distributions with higher order of nonlinearities. It is possible to notice good agreement of theoretical formulas and the simulation results for SNR greater or equal than 0 dB. For SNR less than 0 dB, the theoretical analysis is no longer valid because the perturbation method, on which the statistical analysis of the estimation was based, is not satisfied enough. Both the SCPF and the PCPF provide a bigger difference between peak magnitude of IFR spectrum and noise level compared to the standard CPF and other time-frequency representations. These features of presented estimators make the detection and estimation more effective. The complete assessment of reliability of CR estimates can be obtained by joint analysis of detection and estimation characteristics. As shown in the paper, the occurrence of the SNR threshold visible on the estimation characteristics results from the detection characteristics. The presented research has also proved that increase of number of slices in the SCPF and PCPF estimators improves the detection efficiency and estimation accuracy.

The proposed approach of detection and parameter estimation of LFM signals offers the reduction of computational cost compared to standard T-F and T-FR distributions and methods involving pattern recognition in time-frequency images. Moreover, the calculation procedure can be carried out in a parallel way for each slice. In this case the total computation time of SCPF and PCPF-based estimators is close to the calculation time of the standard CPF.

## Figures and Tables

**Table 1:** Comparison of the SCPF-based estimator with other methods.

$a_{2}$ Estimation Method	SNR Threshold [dB]	Signal Parameters	Publication
HAF	11	P=4, N=256	[34]
HAF	17	P=5, N=256	[34]
PHAF	5	P=4, N=256	[34]
PHAF	9	P=5, N=256	[34]
STFT	3	P=4, N=256	[19]
STFT	16	P=5, N=256	[19]
QML	0	P=4, N=256	[34]
QML	−1	P=5, N=256	[34]
Complex STFT	0	P=2, N=250	[26]
WHT	5	P=2, N=16	[15]
HOCPF-WD	1	P=6, N=128	[30]
ICPF	−6 (256 slices) estimation	P=2, N=256	[29]
ICPF	−3 (64 slices) detection	P=2, N=64	[29]
Proposed SCPF	−8 (31 slices) estimation	P=2, N=255	Proposed method
Proposed SCPF	−8 (31 slices) detection	P=2, N=255	Proposed method
Proposed SCPF	−6 (31 slices) estimation	P=2, N=63	Proposed method
Proposed SCPF	−6 (31 slices) detection	P=2, N=63	Proposed method
Proposed SCPF	−12 (31 slices) estimation	P=2, N=1023	Proposed method
Proposed SCPF	−12 (31 slices) detection	P=2, N=1023	Proposed method
